# Transient Migration of Large Numbers of CD14^++^ CD16^+^ Monocytes to the Draining Lymph Node after Onset of Inflammation

**DOI:** 10.3389/fimmu.2016.00322

**Published:** 2016-08-29

**Authors:** Hege Lund, Preben Boysen, Caroline Piercey Åkesson, Anna Monika Lewandowska-Sabat, Anne K. Storset

**Affiliations:** ^1^Department of Food Safety and Infection Biology, Norwegian University of Life Sciences, Oslo, Norway; ^2^Department of Basic Sciences and Aquatic Medicine, Norwegian University of Life Sciences, Oslo, Norway

**Keywords:** monocytes, inflammation, lymph node, migration, pro-inflammatory cytokines

## Abstract

The dynamics of skin-draining cells following infection or vaccination provide important insight into the initiation of immune responses. In this study, the local recruitment and activation of immune cells in draining lymph nodes (LNs) was studied in calves in an adjuvant-induced inflammation. A transient but remarkably strong recruitment of monocytes was demonstrated after onset of inflammation, constituting up to 41% of live cells in the draining LNs after 24 h. Numerous CD14^+^ cells were visualized in subcutaneous tissues and draining LNs, and the majority of these cells did not express dendritic cell-associated markers CD205 and CD11c. In the LNs, recruited cells were predominately of a CD14^++^ and CD16^+^ phenotype, consistent with an intermediate monocyte subset characterized to possess a high inflammatory potential. Moreover, monocytes from the draining LN showed a high expression of genes coding for pro-inflammatory cytokines, including *IL-1*β, *IL-6, TNFa*, and *TGF*β. Shortly after their appearance in the LN cortical areas, the monocytes had moved into the medulla followed by an increase in peripheral blood. In conclusion, this study provides novel information on *in vivo* monocyte recruitment and migration after onset of inflammation.

## Introduction

A protective immune response to infection or vaccination is dependent on the recruitment of immune cells to the inflamed tissue, followed by their activation and the subsequent movement of cells and antigens to the draining lymph node (LN). In this respect, the migration of antigen-loaded dendritic cells (DCs) and recirculating lymphocytes has been extensively studied [reviewed by Girard et al. (1)].

Circulating monocytes are traditionally regarded as short-lived precursors of tissue macrophages and monocyte-derived DCs (moDCs), recruited to tissues for supplementation of these cell populations during homeostasis and for expansion during inflammation (2, 3). The conventional view is that DCs rather than monocytes subsequently migrate from the inflamed tissues to LNs (1). However, monocytes display an array of pattern recognition receptors, which enables them to react to danger and pathogenic stimuli and produce cytokines, and recent studies indicate that monocytes may have distinct effector functions of their own, including the transport and presentation of antigen (4–9), functions that were previously designated to DCs only.

In humans, monocytes can be classified into subsets based on their expression of the lipopolysaccharide (LPS) receptor CD14 and the FcγIIIR CD16 (10, 11). Classical monocytes are CD16-negative and form the major population in blood. The minor CD16-positive monocyte population can be further subdivided into a CD14^+^ CD16^++^ non-classical subset and a third less well-defined CD14^++^ CD16^+^ intermediate subset, suggested to represent a transitional subset between the classical and non-classical monocytes (12). Whereas the classical and intermediate subsets possess pro-inflammatory properties, the non-classical subset may serve a patrolling function (10, 13, 14). However, the precise roles of the different monocyte subsets, and in particular intermediate monocytes, are not well defined neither in the steady state nor under different inflammatory conditions. The most realistic approach to reach experimental evidence for such roles is *in vivo* animal studies. Circulating bovine monocytes have recently been described as phenotypically similar to humans, as the same three subsets based on CD14 and CD16 expression have been recognized in cattle (15–17). Thus, the use of the cow as an animal model may overcome some of the challenges of the large phenotypical differences between mouse and human monocytes (18).

The trafficking of monocytes is mediated by a multitude of chemokine receptors, and the different subsets show different receptor expression profiles. Especially the CC-chemokine receptor 2 (CCR2) and the CX_3_C-chemokine receptor 1 (CX_3_CR1) can be applied to distinguish between different subsets in humans and mice (11, 19, 20). Human classical monocytes express high levels of CCR2 and low levels of CX_3_CR1 and are accordingly poised to traffic to sites of infection and inflammation, whereas non-classical monocytes have a high expression of CX_3_CR1 (19, 21, 22). As an intermediate subset, CD14^++^ CD16^+^ monocytes most likely express both receptors. However, since the majority of studies on this topic refer to CD14^+^ versus CD16^+^ monocytes or to mouse monocyte subsets, this is not fully resolved. Adhesion molecules, such as L-selectin (CD62L) are also important in monocyte trafficking, enabling their adhesion to endothelium and transmigration into tissue.

The majority of studies describing phenotypical and functional characteristics of myeloid cells are based on *in vitro* differentiated blood-derived monocytes. The aim of this study was to characterize the *in vivo* recruitment and activation of immune cells in inflamed tissue and the draining LN, using a bovine model. For this purpose, we used a saponin-based adjuvant, which has been shown to induce both humoral and cellular immunity (23), and an efficient induction of leukocyte recruitment to the draining LN of mice (24, 25). We show here that the induction of inflammation in calves resulted in a surprisingly potent recruitment of cells to the draining LN, dominated by CD14^++^ CD16^+^ monocytes. The migrating cells retained their monocytic character rather than differentiating into moDCs, and showed a high expression of genes coding for pro-inflammatory cytokines. Altogether, these results provide novel information on the phenotype and functional capacity of monocytes after the onset of inflammation, and challenge the conventional view of monocyte trafficking *in vivo*.

## Materials and Methods

### Animals and Experimental Design

Animals were clinically healthy Norwegian Red dairy (NRF) calves of both sexes, of 8–9 weeks of age, raised in commercial Norwegian dairy farms. The first trial included 14 animals distributed into four experimental groups and kept in separate pens: 6 calves served as non-injected controls, whereas 8 calves were injected with 500 μg Matrix-Q™ (a kind gift from Novavax AB, Uppsala, Sweden). The adjuvant was suspended in 2 ml sterile Hanks’ balanced salt solution (Gibco, Life Technologies) prior to injection and administered as a single subcutaneous dose in the left posterior flank region, in an area drained by the subiliac LN. The contralateral skin and subiliac LN were untreated. A second trial included four new calves, which received the same treatment as in the first trial in addition to an injection with a 10-fold lower dose (50 μg) of Matrix-Q™ in the left neck region, in an area drained by the superficial cervical LN. The results presented herein refer to the first trial, unless otherwise stated.

Calves were given acidified milk or milk replacer, calf concentrate, water ad lib and access to straw, and the health status of the animals was examined twice daily. All experimental procedures were conducted in accordance with the laws and regulations controlling experiments using live animals in Norway: the Norwegian Animal Welfare Act of 28 December 2009 and the Norwegian Regulation on Animal Experimentation of 15 January 1996. The study was approved by the Norwegian Animal Research Authority (Norwegian Food Safety Authority).

### Tissue Collection and Preparation

EDTA blood samples were collected prior to adjuvant injection (pre-injected samples), and from the experimental groups at 24 h (*n* = 3), 48 h (*n* = 3), and 96 h (*n* = 2) post-injection in the first trial, and at 24 h (*n* = 4) post-injection in the second trial. The 96 h group was also sampled for blood at 72 h. Hematological differential counting was performed on EDTA blood (Advia^®^ 2120 Hematology System, Siemens AG, Erlangen, Germany). Bovine peripheral blood mononuclear cells (PBMCs) were isolated by density gradient centrifugation (2210 × *g*, 30 min) on lymphoprep (Axis-Shield, Norway), and either analyzed immediately by flow cytometry (FCM), or added freezing medium (Recovery™ cell culture freezing medium, Gibco) for further storage in liquid nitrogen.

Subiliac LNs from calves in the non-injected controls (*n* = 6) were collected at a conventional slaughterhouse. Injected calves were stunned by a captive bolt pistol and exsanguinated, and subjected to post-mortem examination at the Norwegian University of Life Sciences. Samples in the first trial were collected at 24, 48, and 96 h post-injection, and included the draining subiliac LN and the contralateral LN. In the second trial, samples were collected at 24 h and also included the draining superficial cervical LN.

Skin with subcutaneous tissue and LNs on the injected side and the contralateral flank were collected and fixed for histology and immunohistochemistry (IHC). Formalin fixed samples were embedded in paraffin wax and prepared by standard procedures before staining with hematoxylin and eosin (HE) for light microscopy. Skin and LN specimens were frozen in chlorodifluoromethane (Isceon™) chilled with liquid nitrogen, and stored at −70°C until further preparation.

Lymph nodes were excised vertically, and the anterior half toward the injection site was subjected to tissue preparation. LN tissue was minced mechanically by scissors in the presence of PBS/EDTA buffer. First, the LN cell suspensions were filtered through a Cell Strainer™ (BD Falcon), second, through a cotton filter pad soaked with PBS/EDTA, and finally washed in PBS/EDTA before direct analysis or freezing as described above.

### Immunohistochemistry

Cryostat sections were cut 7 μm in thickness, mounted onto poly-lysine-coated slides and stored at −70°C before use. The sections were air dried at room temperature (RT), fixed in ice cold acetone, and finally rinsed and rehydrated in PBS pH 7.3. An indirect immunoperoxidase staining technique was performed on the sections by using an avidin–biotin complex method with the aid of a commercial kit (Vector Laboratories, Burlingame, CA, USA). To avoid non-specific binding of the biotinylated antibody, a blocking solution containing normal horse serum diluted 1:50 in 5% BSA/TBS and avidin diluted 1:6 was applied to the sections for 20 min at RT. The blocking solution was carefully tapped off the slides. Antibodies diluted in 1% BSA/TBS were added to the slides and the slides incubated overnight at 4°C. The subsequent day, the slides were washed carefully in PBS 3 × 5 min, and biotinylated horse anti-mouse IgG was diluted 1:100 in 1% BSA/TBS and added to the slides for 30 min at RT. The slides were washed carefully in PBS 3 × 5 min. Endogenous peroxidase was inhibited by treatment with 1% H_2_O_2_ in methanol for 15 min, followed by rinsing in PBS for 3 × 5 min. The avidin–biotin–horse radish peroxidase complex solution was prepared at least 30 min prior to use, according to kit instructions. The sections were incubated with the complex solution for 30 min. All incubations were done in a slowly rotating humid chamber at RT. Peroxidase activity was visualized by incubation with Imm Pact AEC peroxidase substrate. The reaction was stopped by rinsing in PBS. Slides were counterstained with Mayer’s hematoxylin for 15 s, rinsed in PBS, and mounted. To control for non-specific binding, all runs included a control section where the primary antibodies were replaced by 1% BSA/TBS.

### Immunofluorescence

Immunofluorescent (IF) staining was performed essentially as previously described (26). Briefly, 7 μm cryostat sections were fixed in acetone and treated with 20% BSA/TBS in order to block non-specific binding. One of the following two mixtures of three primary antibodies were added to the sections: mouse anti-human CD14 (Tük4, IgG2a), mouse anti-bovine CD205 (MCA1651G, IgG2b) (both AbD Serotec), and mouse anti-bovine CD11c (BAQ153A, IgM) (VMRD), or mouse anti-human CD14, mouse anti-ovine CD21 (DU2-74-25, IgG2b) (a kind gift from W. Hein), and polyclonal rabbit anti-ki67 (Abcam) all used at previously determined optimal dilutions. Secondary antibodies were isotype-specific Alexa Fluor (350, 594, and 488) (Molecular Probes, Inc., USA). All incubations were done in a slowly rotating humid chamber for 1 h at RT. Slides were mounted in polyvinyl alcohol and stored at 4°C until examination. Control sections were included, replacing the primary antibody with 1% BSA/TBS, and replacing the secondary antibody with an irrelevant antibody. All tissue sections were examined in a Carl Zeiss Axio Imager M2 microscope equipped with a conventional camera (Axiocam HRc Rev. 3) and fluorescence camera (Axiocam HRm Rev. 3).

### Flow Cytometry

Flow cytometry analysis was performed on fresh or previously frozen LN cell suspensions or PBMCs. Cells were first stained with LIVE/DEAD^®^ Fixable aqua or yellow dead cell stain kit (Invitrogen), following the manufacturer’s instructions. Primary unconjugated monoclonal antibodies applied in the current study were mouse anti-bovine and against the following molecules: CD14 (CAM36A, IgG1), CD3 (MM1A, IgG1), CD62L (BAQ92A, IgG1), granulocyte marker (CH138A, IgM), CD11b (MM12A, IgG1) (all Monoclonal Antibody Center, Washington State University, USA), anti-ovine CD21 (DU2-74-25, IgG2b), and mouse anti-bovine CD205 (MCA1651G, IgG2b) (AbD Serotec/BioRad). Directly conjugated antibodies were cross-reactive anti-human CD16-FITC (KD1, IgG2a) and CD14-Pacific blue (Tük4, IgG2a) (both AbD Serotec/BioRad). Secondary isotype-specific reagents were either PE-conjugated or APC-conjugated (Southern Biotech, Birmingham, USA), or Alexa Fluor 488-or 647-conjugated (Molecular Probes/Life Technologies) polyclonal goat-anti-mouse antibodies, or PerCP- eFluor 710 conjugated rat anti-mouse monoclonal antibody (eBioscience/Affymetrix). All antibodies were used at previously determined optimal concentrations. FCM was performed with a 3-laser Gallios flowcytometer (Beckman Coulter), and gating based on staining with secondary antibodies only or isotype controls. Data were analyzed using the Kaluza software (Beckman Coulter).

### Isolation of CD14^+^ Cells and RT-qPCR Analysis

Cell suspensions from the draining subiliac LNs collected at the second trial were used for isolation of CD14^+^ cells and RT-qPCR analysis. Cell suspensions were either snap frozen (*n* = 3) or used further for cell isolation (*n* = 4), as previously described (27). Briefly, CD14^+^ cells were extracted by positive selection of monocyte differentiation antigen CD14 using anti-human CD14 MACS Microbeads (coated with mAb clone Tük4) (Miltenyi Biotec GmbH, Bergisch Gladbach, Germany), according to the manufacturer’s instructions. Purity of selected cells was verified by FCM, and was found to be in the range of 95–98%. Isolated CD14^+^ cells were snap frozen in liquid nitrogen and transferred to −70°C for further storage.

Peripheral blood mononuclear cells used for CD14^+^ baseline isolation were obtained from healthy NRF calves of 8–9 weeks of age (*n* = 3). Total RNA was isolated from LN cell suspensions (6 × 10^6^ cells), LN CD14^+^ cells (16 × 10^6^ to 2.7 × 10^6^ cells) and blood CD14^+^ baseline cells (6 × 10^6^ cells), using the MirVANA isolation kit (Ambion, Austin, TX, USA) following the manufacturer’s instructions. All RNA samples were treated with amplification grade DNase I (Invitrogen) to remove any traces of genomic DNA, and RNA concentration and quality was measured using NanoDrop 1000 (Thermo Fisher Scientific, Wilmington, USA) and 2100 BioAnalyzer (Agilent Technologies, Palo Alto, USA), respectively. All samples had a RNA integrity number (RIN) above 8.7 (except one where RIN = 6.6) and an OD A260/A280 ratio of ≥2.0. A total of 200 ng of RNA was used for cDNA synthesis reaction using Tetro cDNA synthesis kit (Nordic BioSite, Norway), and 10 ng was used in qPCR in triplicate per sample using Express SYBR GreenER SuperMix with premixed ROX (Invitrogen) according to the manufacturer’s recommendations. Transcript levels were analyzed using a 7900HT Fast Real-Time PCR System (Applied Biosystems) and the standard cycling program: 50°C for 2 min, 95°C for 2 min, 40 cycles of 95°C for 15 s, and 60°C for 1 min, and the melting curve analyses were applied. Gene-specific primers were from either literature or designed using Primer3 ver. 0.4.0 (28). The transcript levels of the following genes were analyzed: *CD14, CD16*α, *IL-1*β, *IL-6, TNF*α, *TGF*β, *IL-12*β, *IL-10, CCR2*, and *CX_3_CR1*. Primer sequences are presented in Table 1. The efficiencies of all primer pairs were tested by template dilution series using pooled cDNA from LN cells suspensions and CD14^+^ baseline cells and were 100% (±10). Negative controls with no added template were included for all primer pairs (no template control), and no RT control reactions for each sample and each primer pair were run in qPCR in order to check for genomic DNA contamination (no RT control). The *peptidylprolyl isomerase A* (*PPIA*) reference gene selected for the current study has been shown to be one of the most stable genes for gene expression studies in cattle macrophages (27) and lymphocytes (29), and in human LPS-stimulated monocytes (30). Initial analysis of the RT-qPCR data was performed using RQ Manager 1.2 (Applied Biosystems). Standard deviation of ≤0.3 per triplicate was accepted. The ΔCt method was used to calculate RT-qPCR data, i.e., ΔCt = Ct_target gene_ − Ct_reference gene_, and normalized gene expression was calculated as 2 ^(−ΔCt)^. Distribution of the data for the expression levels for each gene was tested by Shapiro–Wilk’s normality test in R (R: A Language and Environment for Statistical Computing, ver. 3.2.4, The R Core Team, 2016). The differences of normalized gene expression levels between CD14^+^ baseline cells and CD14^+^ cells from LN for each gene were tested using either unpaired *t*-test (for normally distributed data) or the unpaired Wilcoxon rank-sum test (for non-normally distributed data) in GraphPad (GraphPad Prism version 7.00 for Windows, GraphPad Software). Statistical significance was assigned at *P* ≤ 0.05.

**Table 1:** **List of primers used for reverse transcription-quantitative PCR (RT-qPCR)**.

Gene symbol, accession no.		Primers (5′ → 3′)	Amp (bp)	Reference
CD14, NM_174008.1		CGATTTCCGTTGTGTCTGC	150	(16)
		TACTGCTTCGGGTTGGTGT		
CD16α, NM_001077402.1	*Low-affinity* FcγIIIR	TGTCTCGTCATTCTTTCTACCTTG	138	(16)
		ACTTTGCCATCCCTCCATTC		
CX3CR1, NM_001102558.2	*CX_3_C-chemokine receptor 1*	TCACCAGAGAGAAAGAGAACGA	108	(16)
		GGAGCAGGAAGCCAAGAAA		
CCR2, NM_001194959	*CC-chemokine receptor 2*	GATGAAGAACCCACCACCAG	118	(16)
		CAAAGATGAAGACCAGCGAGTAG		
TGFβ1, NM_001166068.1	*Transforming growth factor beta 1*	CAATTCCTGGCGCTACCTCA	121	Primer 3
		GCCCTCTATTTCCTCTCTGCG		
IL-1β, NM_174093.1	*Interleukin-1 beta*	AAAAATCCCTGGTGCTGGCT	89	Primer 3
		CATGCAGAACACCACTTCTCG		
IL-6, NM_173923.2	*Interleukin-6*	CCTGAAGCAAAAGATCGCAGA	97	Primer 3
		TGCGTTCTTTACCCACTCGT		
IL-10, NM_174088.1	*Interleukin-10*	TATCCACTTGCCAACCAGCC	152	Primer 3
		GGCAACCCAGGTAACCCTTA		
IL-12β, NM_174356.1	*Interleukin-12 subunit beta*	GAGGTCGTGGTAGAAGCTGT	87	Primer 3
		TGGGTCTGGTTTGATGATGTCC		
TNFα, NM_173966.3	*Tumor necrosis factor alpha*	TCTTCTCAAGCCTCAAGTAACAAG	103	(27)
		CCATGAGGGCATTGGCATAC		

*Amp, amplicon*.

### Statistics

Flow cytometry data were analyzed in the JMP Pro 12 statistical software (SAS Institute). Differences between groups consisting of different individuals were assessed by the Wilcoxon rank-sum test, and are indicated by *. Differences between groups consisting of the same individuals were assessed by the paired *t*-test, and are indicated by ^#^. Statistical significance was assigned at *P* < 0.05.

## Results

### Adjuvant Injection Led to a Strong Recruitment of Monocytes

A saponin-based adjuvant was injected subcutaneously in the flank region of calves. Calves were euthanized at different time points following injection, and blood as well as tissues at the injection and contralateral sites, including skin, draining, and contralateral LNs, were subjected to pathological and immunological analyses. On macroscopic evaluation, the injection site was characterized by a subcutaneous edema, and the draining LN was found to be two- to threefold enlarged (not shown). These changes were most pronounced at 24 h post-injection. Skin and LNs from the contralateral side did not show these changes.

**Figure 1:**
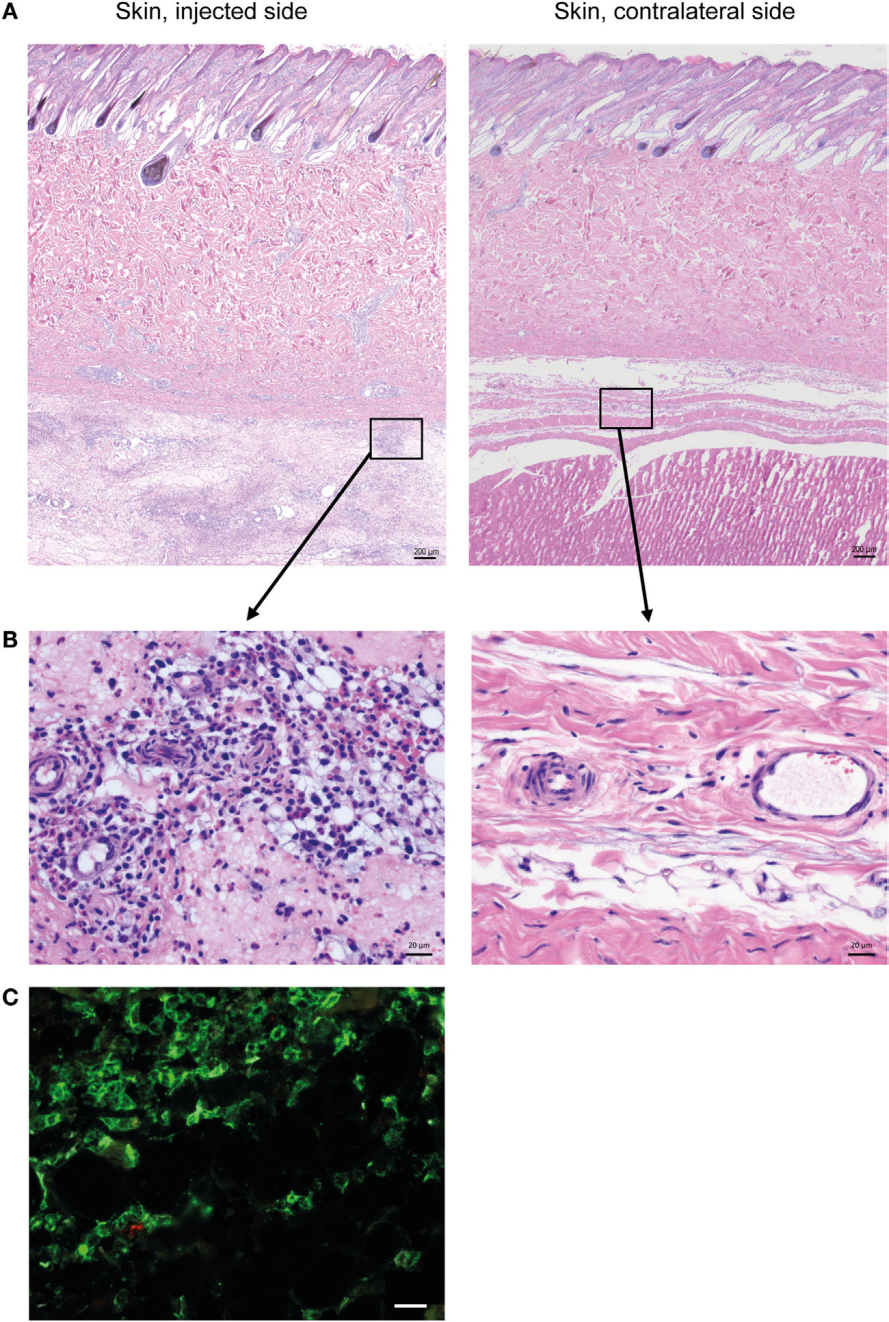
**Cellular recruitment to skin and subcutaneous tissues**. **(A)** HE stained sections of skin with subcutaneous tissue from the side injected with adjuvant and the contralateral side, at 24 h post-injection. Scale bars: 200 μm. **(B)** Enlargement of outlined areas in A, as indicated. Scale bars: 20 μm. **(C)** Immunofluorescent labeling of subcutaneous tissue on the injected side with antibody against CD14 (green). Scale bar: 20 μm.

Histopathological evaluation of the skin on the injected side revealed a diffuse, locally extensive, and moderate-to-severe inflammation in deeper cutaneous and subcutaneous tissues (Figure 1A), consisting of an infiltration of neutrophils, lymphocytes, and large monocyte-like cells (Figure 1B). A substantial amount of the inflammatory infiltrate consisted of CD14^+^ cells (Figure 1C).

**Figure 2:**
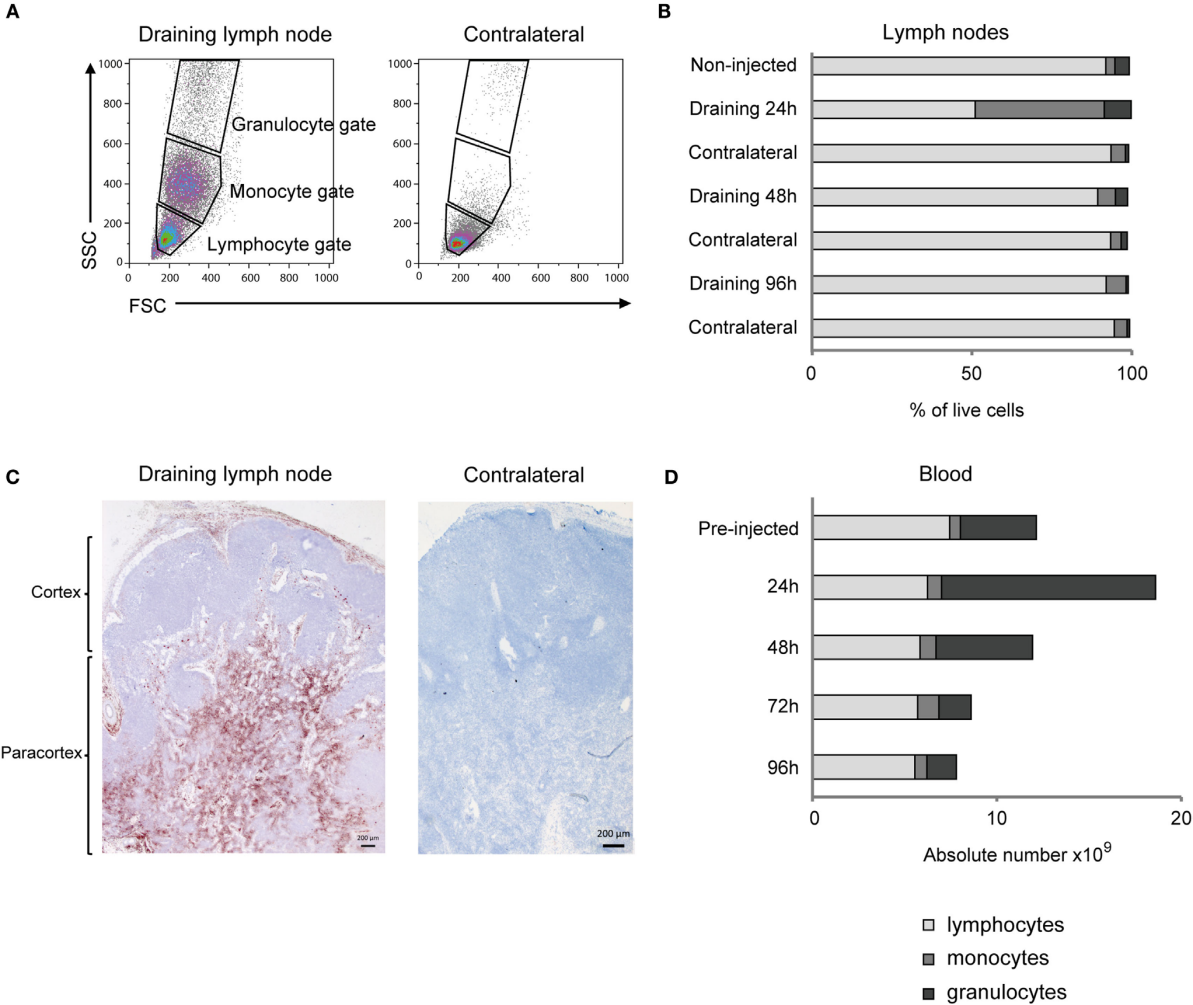
**Cellular recruitment to lymph nodes (LN) and peripheral blood**. **(A)** LN cells were prepared for FCM analysis and gated on forward/side scatter (FSC/SSC) characteristics. Plots from one representative animal are presented. Panels illustrate the gating of lymphocytes, monocytes, and granulocytes as indicated and in the draining LN (left) and the contralateral LN (right), at 24 h post-injection. **(B)** Percentages of major immune cell populations in LNs, based on the gating strategy in A. Horizontal stacked bars show mean percentages of lymphocytes (gray), monocytes (dark gray), and granulocytes (black) of the total live cell population in non-injected animals (*n* = 6) and at different time points after adjuvant injection (*n* = 2–3). **(C)** IHC labeling of draining and contralateral LNs at 24 h post-injection with antibody against CD14. Different regions of the LN are indicated. **(D)** Cellular differential counts in peripheral blood. Horizontal stacked bars show mean absolute numbers (×10^9^) of lymphocytes (gray), monocytes (dark gray), and granulocytes (black) at pre-injection and at different time points after adjuvant injection.

Flow cytometry analysis of cells from the draining LN revealed a distinct appearance of numerous cells in the monocyte gate at 24 h post-injection, which were only scarcely present in the contralateral LN (Figures 2A,B). Also the relative percentage of cells in the granulocyte gate was increased, while the overall lymphocyte population was reduced at this time point. Detailed results are presented in Table S1 in Supplementary Material. IHC staining of the draining LN demonstrated that numerous CD14^+^ cells were present in the subcapsular and peri-trabecular sinuses, and in the T-cell zones of the cortex (Figure 2C).

There was a marked and transient increase in the absolute number of granulocytes in peripheral blood, peaking at 24 h post-injection, with a mean fold increase of 2.8 from pre-injected levels (Figure 2D). The increase in the absolute number of monocytes in blood was less evident and came later, peaking to a double level at 72 h post-injection. Detailed results are presented in Table S2 in Supplementary Material.

### Recruited Monocytes Were CD14^++^ CD16^+^

**Figure 3:**
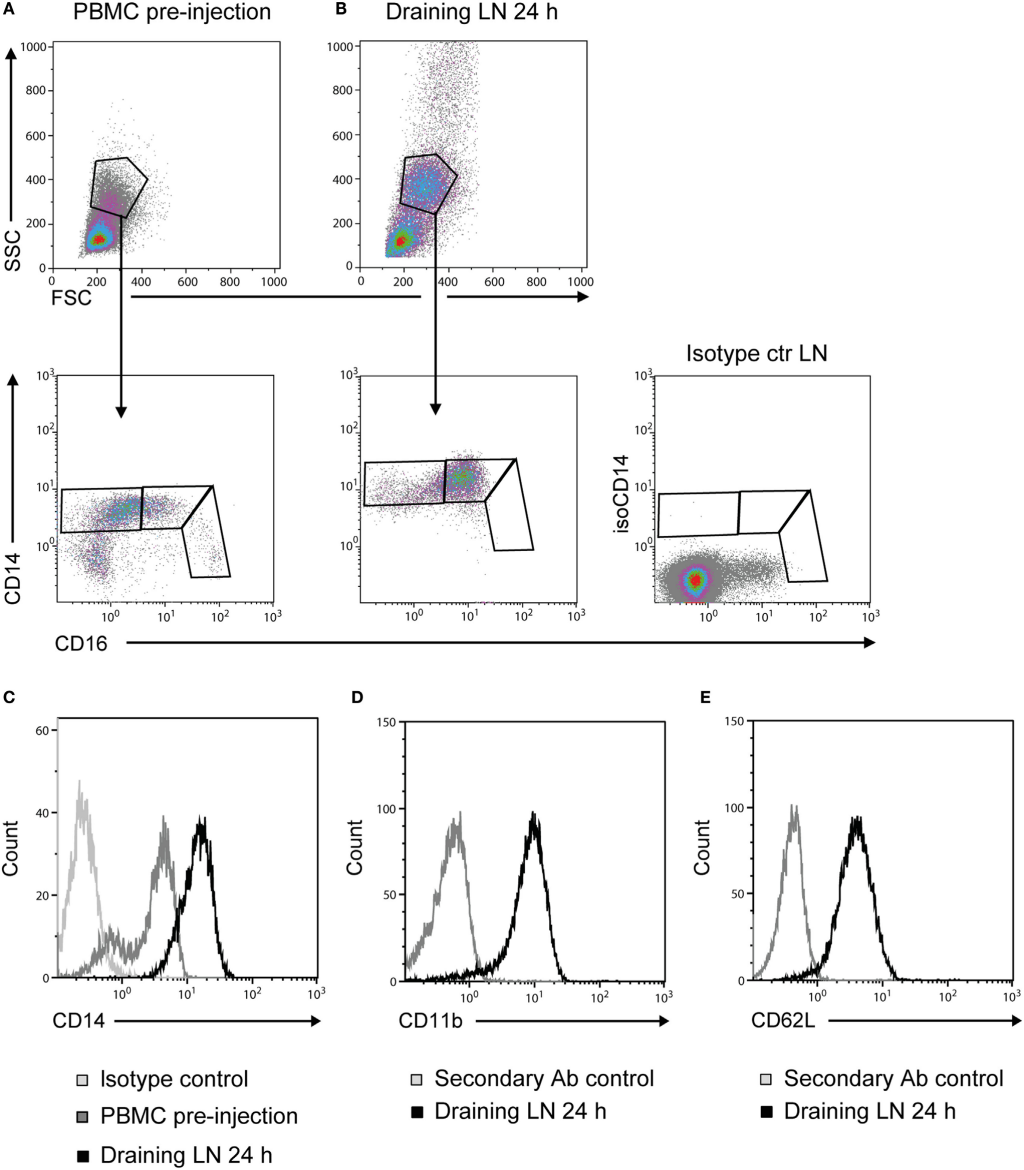
**Phenotype of recruited monocytes**. Density plots of live cells (upper panels) from PBMC pre-injection **(A)** and the draining LN at 24 h post-injection **(B)**. Plots from one representative animal are presented. Monocytes were further gated into subsets based on their expression of CD14 and CD16 (lower panels). Isotype control for CD14 on live cells from LN is shown far right. **(C)** CD14 expression on cells from the monocyte gate [as gated in **(A,B)**]. Histograms show the isotype control for CD14 (light gray line), PBMC baseline (gray line), and draining LN at 24 h (black line). **(D)** CD11b expression and **(E)** CD62L expression on CD14^++^ cells. Histograms show the secondary Ab control (gray line) and the draining LN at 24 h (black line).

Prior to adjuvant injection, monocytes from PBMC could be divided into three different subsets based on the expression of CD14 and CD16 (Figure 3A), coherent with previous reports (16, 17). Monocytes recruited to the draining LNs at 24 h post-injection were of an essentially homogeneous CD14^++^ CD16^+^ phenotype (Figure 3B). Moreover, the intensity of CD14 expression on these LN monocytes was increased in comparison to monocytes from PBMC (Figure 3C). CD14^++^ cells from draining LNs were CD11b^+^ (Figure 3D) and CD62L^+^ (Figure 3E). Monocyte-gated cells did not express the granulocyte antigen (CH138A) or the T-cell marker CD3 in FCM, both confirmed to be present on cells in the respective granulocyte and lymphocyte gates (not shown).

### Monocytes Were Transiently Present in the LN Cortex and Migrated via the Medulla to Blood

**Figure 4:**
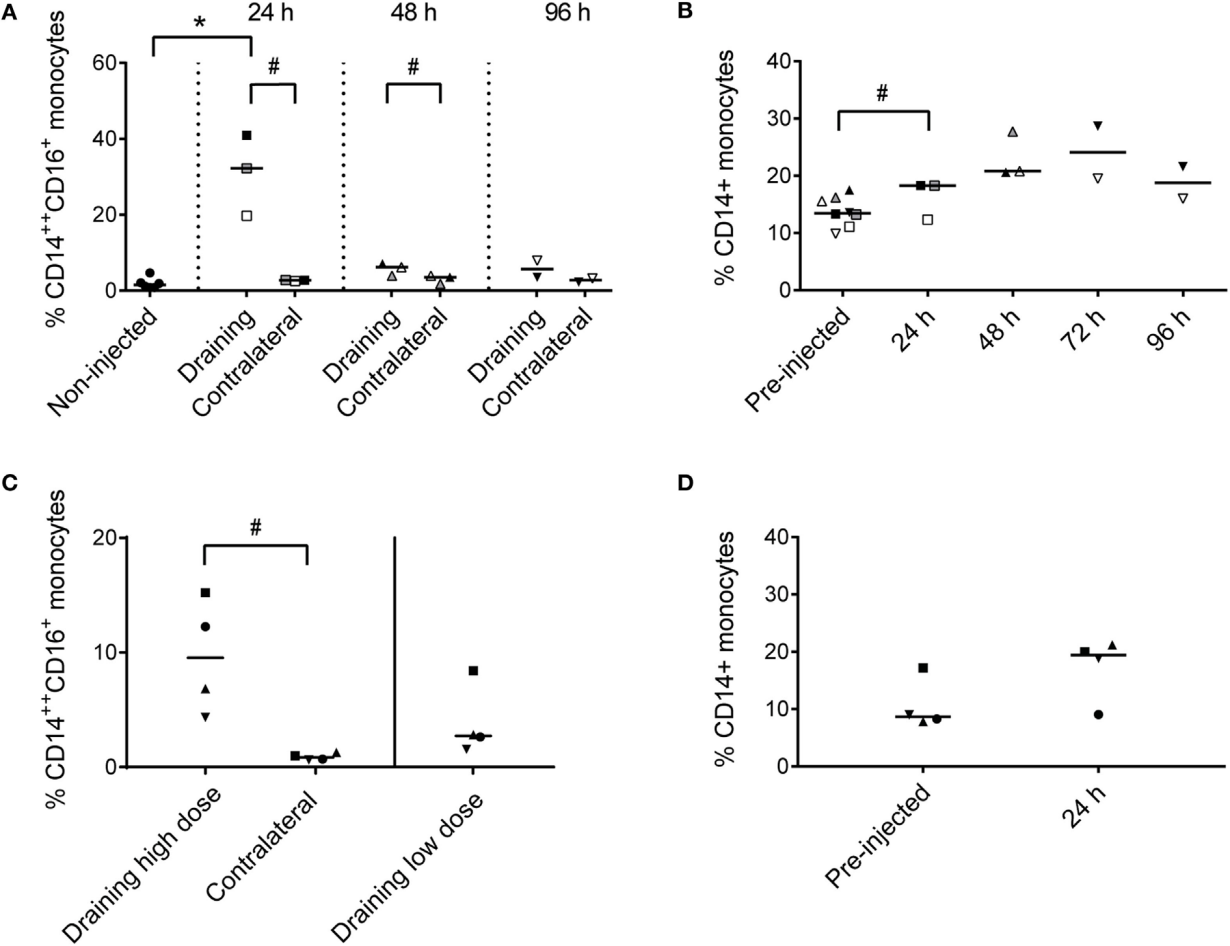
**Percentages of monocytes in LNs and PBMC**. **(A)** Percentages of CD14^++^ CD16^+^ monocytes of total live cells in LNs of non-injected animals, and in draining and contralateral LNs of injected animals at 24, 48, and 96 h post-injection. Symbols represent individual animals and the median value within each group is depicted as a line. Statistical significant differences between injected and non-injected groups using the Wilcoxon rank-sum test are indicated as *P < 0.05. Statistical significant differences between groups consisting of the same individuals (identical symbols) using the paired *t*-test are indicated as ^#^*P* < 0.05. **(B)** Percentage of CD14^+^ monocytes of live cells in PBMC at pre-injection, and at 24, 48, 72, and 96 h post-injection. Symbols and statistics as in **(A)** (^#^*P* < 0.05). **(C)** Percentage of CD14^++^ CD16^+^ monocytes of live cells in the draining high dose (subiliac) LN and the contralateral LN, and in the draining low-dose (superficial cervical) LN. Symbols and statistics as in A (^#^*P* < 0.05). **(D)** Percentage of CD14^+^ monocytes of total live cells in PBMC at pre-injection and after 24 h. Symbols and statistics as in A.

Recruited CD14^++^ CD16^+^ cells constituted 20–41% of live cells in the draining subiliac LN 24 h after adjuvant injection (Figure 4A). In PBMC, CD14^+^ monocytes tended to increase in percentage later; measurably already at 24 h but apparently peaking in the two consecutive days (Figure 4B).

In the second trial, the recruitment of CD14^++^ CD16^+^ monocytes to the draining subiliac LN was again demonstrated, but in lower numbers (Figure 4C). The second trial also included an injection of a 10-fold lower dose of adjuvant in the neck region, drained by the superficial cervical LN, leading to reduced recruitment of monocytes. Like in the previous trial, an increase of CD14^+^ monocytes was demonstrated in blood after 24 h (Figure 4D).

**Figure 5:**
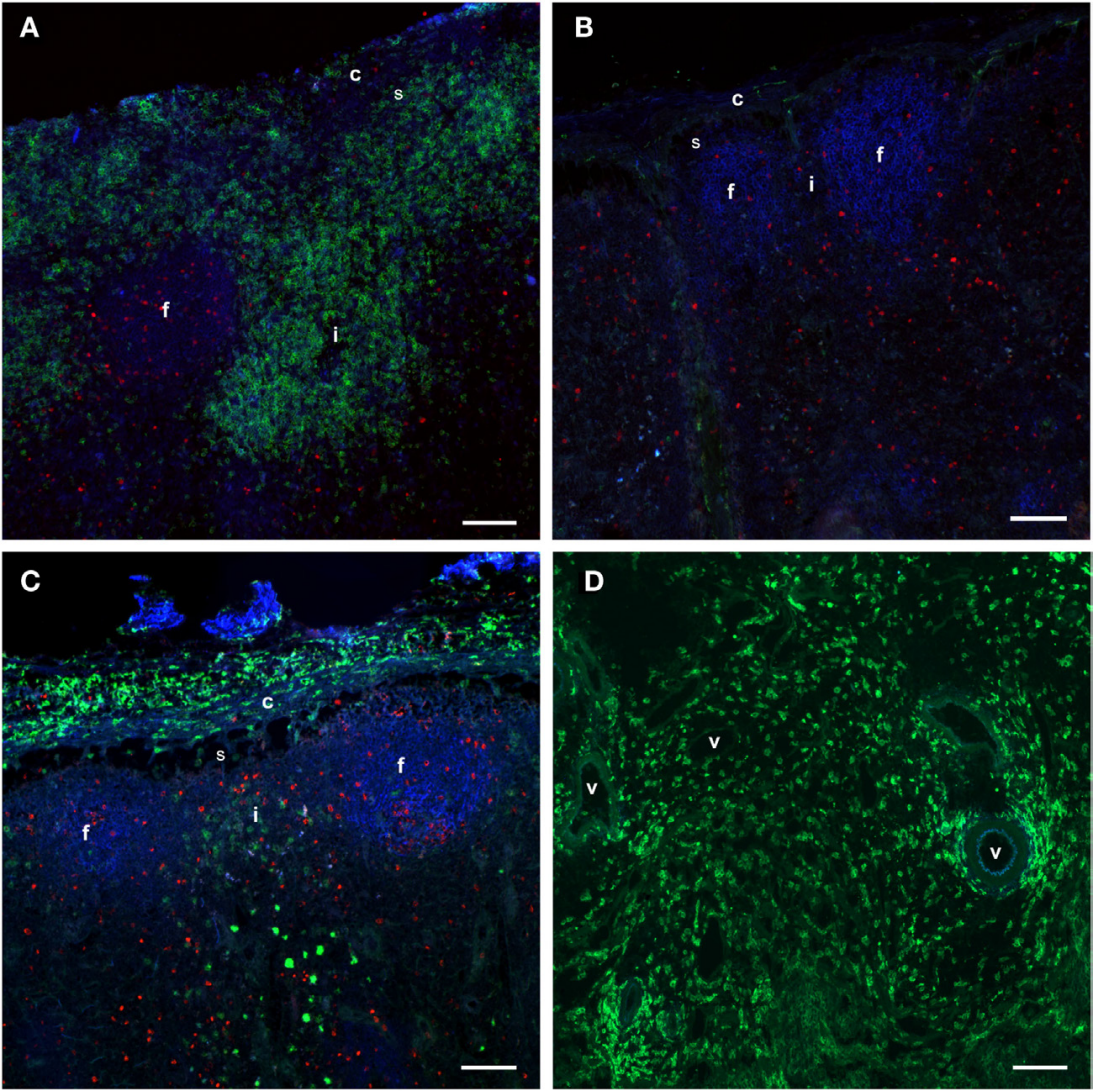
**Distribution of monocytes in the LNs**. Immunofluorescent labeling of LNs with antibodies against CD14 (green), CD21 (blue), and Ki67 (red). CD21 stains the LN follicles. **(A)** CD14^+^ cells were present in the capsule, subcapsular sinus, peri-trabecular sinus, and interfollicular areas of the draining LN at 24 h post-injection. **(B)** The contralateral LN was mainly devoid of CD14^+^ cells. Note the empty sub capsular sinus and trabecular sinus areas, as opposed to the infiltration in **(A)**. **(C)** CD14^+^ cells were abundant in the capsule, but were decreased in numbers in the sinus and the cortex at 48 h post-injection. **(D)** CD14^+^ cells were present in the medulla of the LN at 48 h post-injection, and particularly around vessels. Follicle (f), interfollicular area (i), capsule (c), sinus area (s), vessel (v). Scale bars: 100 μm.

The transient presence of monocytes in the draining LN was also visualized by IF staining. A high number of CD14^+^ cells were present in the draining LN at 24 h post-injection (Figure 5A), but not in the contralateral LN (Figure 5B). Monocytes did not express Ki67, indicating that they were not in active proliferation after entry to the LNs. After 48 h, monocytes had decreased in number in the cortex of the draining LN (Figure 5C), but were present in moderate to large amounts in the medulla, including the area around efferent lymph vessels (Figure 5D). In the second trial, a similar monocyte recruitment was observed to the LN cortex, but in lower numbers, whereas more monocytes were present in the medulla already at 24 h, indicating an earlier onset or a faster migration through the lymphoid tissue than observed in the first trial (not shown).

Taken together, these findings indicated that an adjuvant injection lead to a strong and transient recruitment of monocytes to the LN cortex, followed by migration into the medullary areas before departure via efferent vessels into the blood.

### Monocytes in Skin and Draining LN Did Not Express DC-Associated Markers

**Figure 6:**
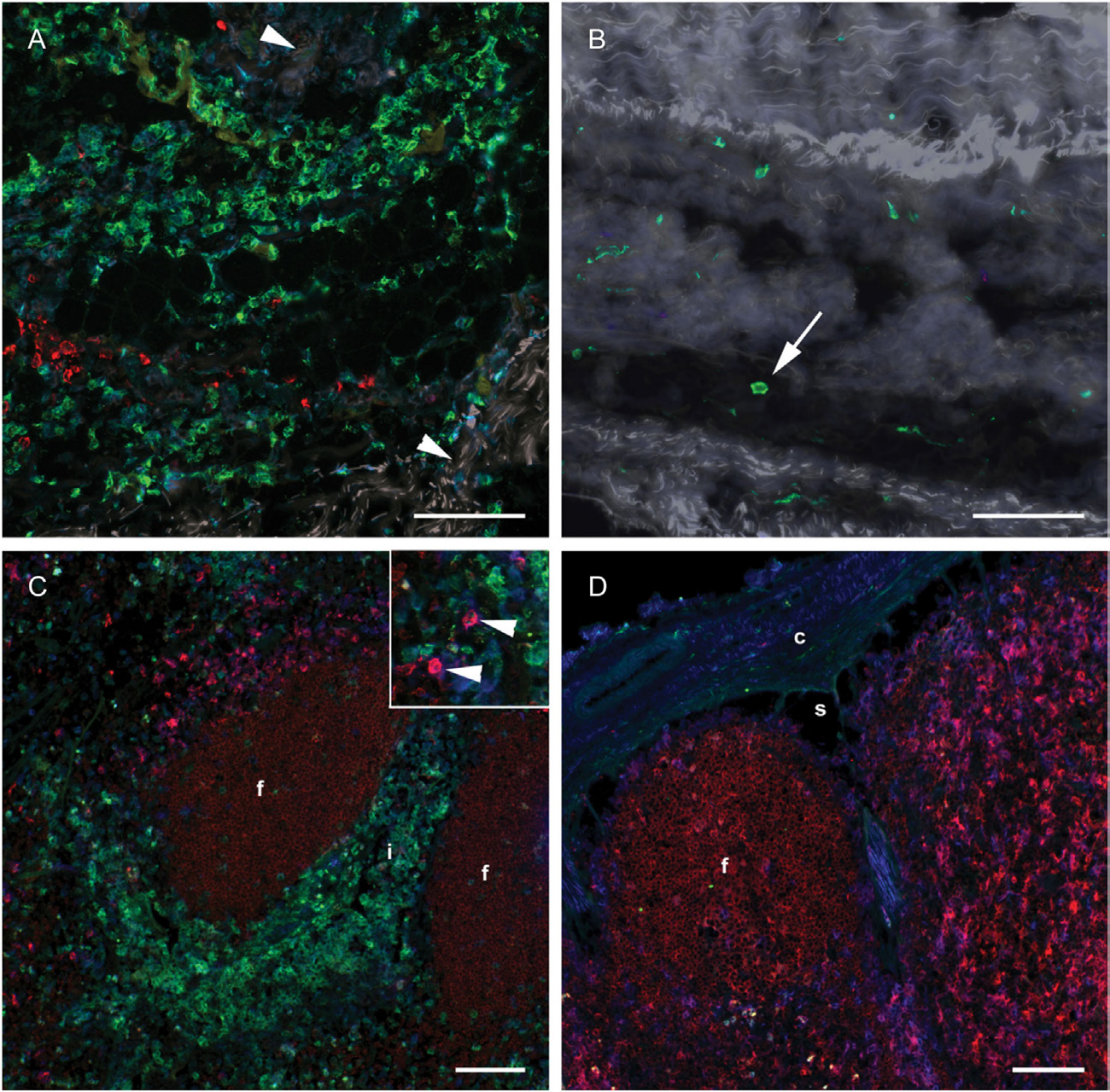
**Distribution of monocyte and DC markers in skin and LNs**. Immunofluorescent labeling of subcutaneous tissues and LNs with antibodies against CD14 (green), CD205 (red) and CD11c (blue), at 24 h post-injection. **(A)** Large numbers of CD14^+^ cells were observed in the subcutis on the injected side. Note the separation of collagen bundles (appears as gray auto fluorescence, arrowheads) due to inflammatory infiltrates. A limited number of CD205^+^ cells were present. **(B)** A few CD14^+^ cells (arrow) were observed on the contralateral side. **(C)** CD14^+^ cells infiltrated the interfollicular areas of the cortex of the draining LN. A moderate amount of CD205^+^ CD11c^+^ DCs were observed (arrow heads in insert). **(D)** CD14^+^ cells were sparsely present in the contralateral LN. CD205^+^ follicles were surrounded by CD11c single labeled and CD11c^+^ CD205^+^ double labeled cells. Follicle (f), interfollicular area (i), capsule (c), sinus area (s). Scale bars: 100 μm.

To investigate whether a differentiation of CD14^+^ cells toward a DC phenotype had taken place in subcutaneous tissues or in the draining LN, IF triple labeling of skin and LN tissue was performed with CD14 and the DC-associated markers CD205 and CD11c. Numerous CD14^+^ monocytes were present in the deep cutis and subcutis on the injected side at 24 h post-injection (Figure 6A). A limited number of CD14^−^ CD205^+^ cells were present, possibly representing macrophages. Very few CD14^+^ cells were present in the skin on the non-injected side (Figure 6B). A moderate amount of CD11c^+^ CD205^+^ cells were observed in both the draining and the contralateral LN at 24 h post-injection, most likely representing conventional LN DCs (Figure 6C, insert, and Figure 6D). These cells did not triple label with the CD14-marker. A minor proportion of CD14^+^ cells in the draining LN were found to be CD11c^+^, while none were CD205^+^. CD205 also labeled cells within the lymphoid follicles, as previously assigned to B cells in cattle (31). In FCM, CD14^++^ cells from the draining LN did not express CD205 (not shown).

### Monocytes in the Draining LN Expressed Genes Coding for Pro-Inflammatory Cytokines and *CCR2*

**Figure 7:**
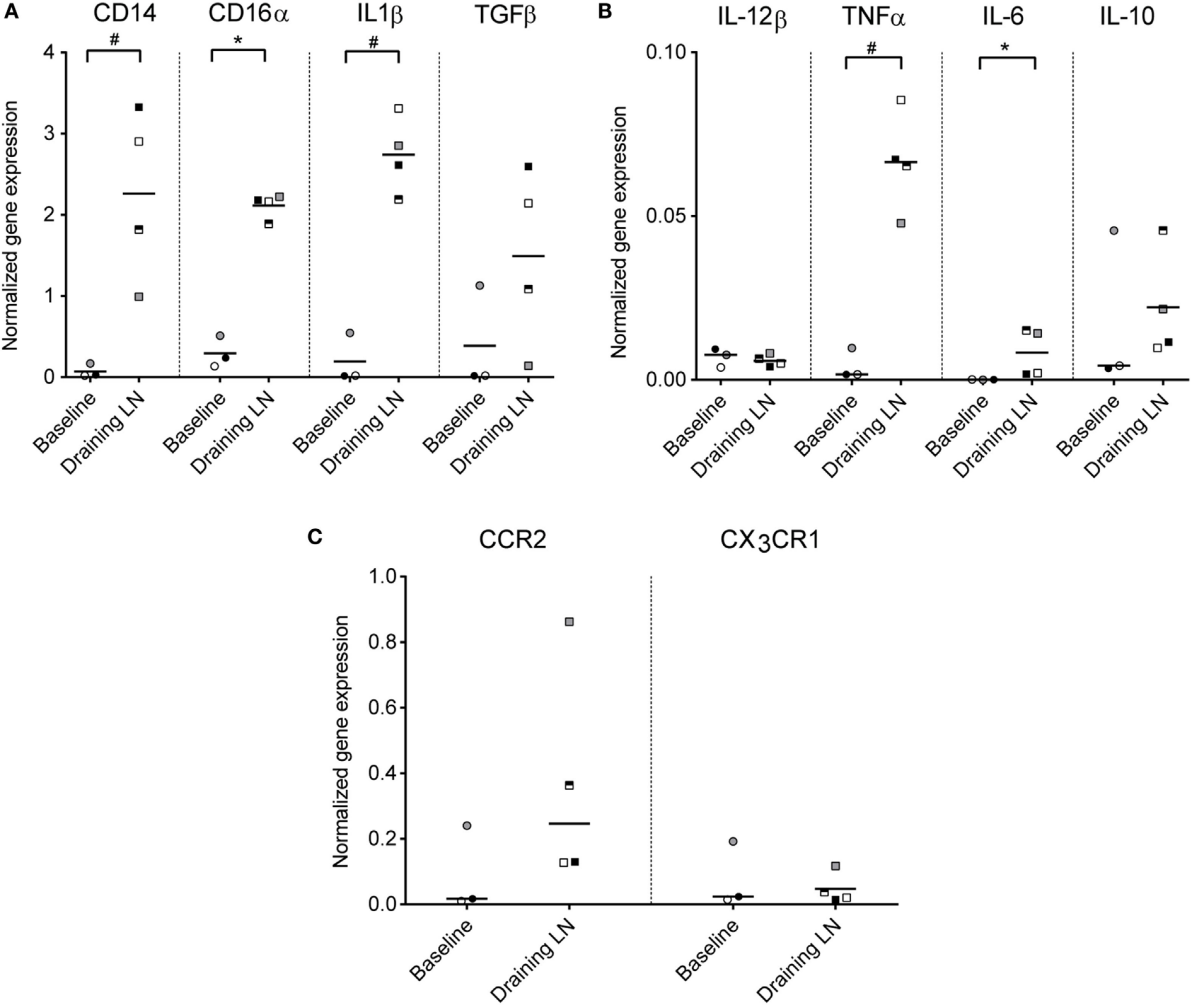
**Gene expression of monocytes from the draining LN**. Gene expression levels (normalized to the reference gene *PPIA*) of CD14^+^ cells isolated from blood (baseline non-injected) and from the draining LN at 24 h post-injection, as analyzed by RT-qPCR. Symbols represent individual animals and the median value within each group is depicted as a line. Statistical significant differences between the two groups using the unpaired *t*-test (for normally distributed data) are indicated as ^#^*P* ≤ 0.05, and the Wilcoxon rank-sum test (for non-normally distributed data) are indicated as *P ≤ 0.05. **(A)** Gene expression levels of *CD14, CD16*α, *IL-1*β, and *TGF*β. **(B)** Gene expression levels of *IL-12*β, *TNF*α, *IL-6, and IL-10*. Note the difference in *y*-axis range from **(A)**. **(C)** Gene expression levels of chemokine receptors *CCR2* and *CX_3_CR1*. Note the difference in *y*-axis range from **(A,B)**.

To investigate the functional capacity of recruited monocytes, CD14^+^ cells from the draining subiliac LNs of injected calves were isolated by positive selection and analyzed by RT-qPCR in the second trial. Baseline gene expression values were obtained from blood CD14^+^ cells isolated from calves of the same age and the same herd and were normalized to a housekeeping gene (*PPIA*). Expression levels of genes encoding for *CD14, CD16*α, *IL-1*β, *IL-6*, and *TNFa* were significantly higher in CD14^+^ cells from draining LNs compared to CD14^+^ cells from blood (*P* ≤ 0.05, Figures 7A,B). *TGF*β also appeared upregulated, although the difference from baseline blood was non-significant in this limited material. No clear difference in expression of *IL-12*β and *IL-10* was found. Of the two chemokine receptors assessed, *CCR2* gene expression level was higher in most CD14^+^ samples from injected animals, but not to a significant degree, likely due to an outlier in the baseline samples (Figure 7C). No difference in expression of the gene coding for the chemokine receptor *CX_3_CR1* was found.

## Discussion

While most studies of cell migration from inflamed tissues to draining LNs have focused on DCs (1), recent studies have shown that also monocytes travel via this route (4, 8, 9, 32). We here demonstrate a potent *in vivo* recruitment of monocytes to the draining LN in a local acute inflammatory situation, and show that these cells upregulate genes for pro-inflammatory cytokines.

Recruited cells were of a uniformly CD14^++^ CD16^+^ phenotype and, thus, phenotypically resembled the intermediate monocyte subset described in humans (11, 20), and recently in cattle (16, 17). However, in contrast to bovine blood monocytes, we found that monocytes recruited *in vivo* to LNs stained more brightly for CD14 (CD14^++^). This phenotype was supported by RT-qPCR findings, showing a high gene expression for both *CD14* and *CD16*α in these cells. CD14^+^ cells isolated from LNs had increased expression of genes associated with induction of inflammation, including *TNF*α, *IL-1*β, *IL-6*, and *TGF*β. This is in line with other reports of intermediate monocytes expanding under different inflammatory conditions [reviewed by Italiani and Boraschi (33) and Ziegler-Heitbrock (34)], and strongly implicates a role for these CD14^++^ CD16^+^ monocytes in inflammatory processes *in vivo*.

Monocytes are believed to mainly differentiate into macrophages or moDCs in the inflamed tissue, after which predominantly moDCs will travel to the draining LN. These moDCs or “inflammatory” DCs should not be confused with conventional DCs that originate from an independent lineage of hematopoietic cells (35), and which have best been described in cattle in the afferent lymph (36–38). We found that the majority of CD14^+^ cells present in subcutaneous tissues and the draining LNs did not express the DC-associated markers CD205 and CD11c. Moreover, unlike moDCs, CD14^++^ cells were CD11b^+^ and CD62L^+^. The majority of studies of monocyte-derived cells in cattle are based on *in vitro* generated cells from blood, which have been shown to downregulate CD14 and CD62L and upregulate CD205 (39–42). In mice, moDCs have been identified based on a high surface expression of CD11c (9, 43), whereas in humans CD11c is considered specific only for those DCs found in lymphoid organs (44, 45). In cattle, both blood monocytes and *in vitro* derived cells express CD11c, and the latter to a lesser degree than the former (16, 41, 42, 46). However, the *in vitro* differentiation of blood monocytes cannot fully recapitulate the differentiation *in vivo*, which may be influenced by a combination of factors in the tissue environment, such as chemokines, cytokines, or administered adjuvant or antigen. Collectively, the overall phenotype indicates that the recruited cells in the present study labeling CD14^++^ CD16^+^ CD11b^+^ CD62L^+^ were monocytes rather than moDCs.

Monocytes were present in the skin and subcutaneous tissue at the injection site and in high numbers in the sinus and the T-cell area of the LN cortex. This is consistent with the possibility of a monocyte migration from the skin to the draining LN via afferent lymph. Indeed, the presence of monocytes in afferent lymph of sheep (4, 47) and rat (32) has been described, and recently, adoptively transferred monocytes were shown to migrate from skin to the draining LN in mice (8, 9). We cannot exclude the possibility that monocytes also entered the LN from blood via high endothelial venules (HEVs), as CD14^++^ cells were strongly CD62L positive, and the recruitment of monocytes from blood across HEVs has been reported (48). However, the predominant route of monocyte trafficking to LNs is thought to be via the afferent lymph (10, 43, 49). We also did not observe any monocytes increase in blood prior to their appearance in LNs. Moreover, we found that the gene expression level of *CCR2* was upregulated in CD14^+^ monocytes from the draining LNs, implicating this chemokine receptor in the recruitment of bovine intermediate monocytes to inflamed tissue.

The recruitment of monocyte to the draining LN was transient, and after 48 h a population of monocytes remained in the medulla only, suggesting an internal migration within the LN toward an exit of these cells through efferent lymph vessels. In support of this notion, we found an increase in CD14^+^ monocytes in PBMC starting around 24 h post-injection. We cannot exclude the possibility that some of these circulating monocytes were recruited from the bone marrow or splenic reservoirs, as expected during an inflammation. Nevertheless, the substantial passage of monocytes through LNs represents a phenomenon that to our knowledge is not well documented in the literature, probably due to its highly transient nature. To get a gross idea of the dose–response effect of the adjuvant, a second trial included an injection of a 10-fold lower adjuvant dose, leading to reduced recruitment of monocytes. However, this was performed at a differing anatomical location, and the evaluation of the efficacy and safety of the given adjuvant in a potential vaccine context warrants additional studies, being outside the scope of the presented study.

A large part of our knowledge on leukocyte recirculation derives from large animal models, and ruminants can serve as excellent *in vivo* models due to their size and the possibility to follow cell migration via lymphatic cannulation (4, 36, 38, 50, 51). More knowledge on the initiation of immune responses in cattle can form a basis for new vaccines in this species, but also be important for understanding processes in mammals at large, including humans. To this end, a combination of methods in experimental post-mortem analyses as presented herein can offer powerful tools for future studies of dynamics and recirculation of immune cells, in the steady state as well as under inflammatory conditions.

## Author Contributions

HL: study design, sample collection and preparation, FCM, data interpretation, and writing of manuscript. PB: study design, FCM, data interpretation, writing, and editing of manuscript. CÅ: study design, IHC and IF stainings, data interpretation, writing, and editing of manuscript. AL-S: study design, sample collection, RT-qPCR analysis, writing, and editing of manuscript. AKS: Study design, data interpretation, writing, and editing of manuscript. All authors approved the final manuscript and are accountable for all aspects of the presented study.

## Conflict of Interest Statement

The authors declare that no financial or commercial conflict of interest exists in relation to the content of this article. The authors have no financial involvement in Novavax AB.

## Abbreviations

CCR2, CC-chemokine receptor 2; CX_3_CR1, CX_3_C-chemokine receptor 1; DCs, dendritic cells; FCM, flow cytometry; HE, hematoxylin and eosin staining; HEVs, high endothelial venules; IF, immunofluorescence; IHC, immunohistochemistry; LN, lymph node; moDCs, monocyte-derived dendritic cells; PBMC, peripheral blood mononuclear cells; *PPIA, peptidylprolyl isomerase A*; RT-qPCR, reverse transcription-quantitative polymerase chain reaction.
