# Supplementary material for: Transient Migration of Large Numbers of CD14++ CD16+ Monocytes to the Draining Lymph Node after Onset of Inflammation
Source: Front Immunol. 2016 Aug 29;7:322. doi: 10.3389/fimmu.2016.00322 (PMC5002921; doi:10.3389/fimmu.2016.00322)
Supplement: Supplementary file 1 [file Table_1.PDF]

		N	% lymphocytes	% monocytes	% granulocytes
	Non-injected	6	92 (88 – 97)	3 (1 - 4)	4 (1 - 9)
24 h	Draining	3	49 (41 - 66)	41 (31 - 49)	8 (8 - 9)
	Contralateral	3	93 (93 - 94)	5 (4 - 5)	1 (1 - 2)
48 h	Draining	3	90 (83 - 95)	6 (3 - 8)	3 (1 - 8)
	Contralateral	3	93 (92 - 94)	3 (3 - 4)	2 (1 - 3)
96 h	Draining	2	(91 - 93)	(5 - 7)	(0 - 1)
	Contralateral	2	(94 - 95)	(4 - 4)	(1 - 1)

Supplementary table 1. Numbers are median percentage and range of cells within the indicated immune cell populations (as gated in Fig. 2A), from live cells in lymph nodes. N=number of animals investigated.
