# Supplementary material for: Transient Migration of Large Numbers of CD14++ CD16+ Monocytes to the Draining Lymph Node after Onset of Inflammation
Source: Front Immunol. 2016 Aug 29;7:322. doi: 10.3389/fimmu.2016.00322 (PMC5002921; doi:10.3389/fimmu.2016.00322)
Supplement: Supplementary file 2 [file Table_2.PDF]

	N	Lymphocytes	Monocytes	Granulocytes
Pre-injected	8	7 (6 - 11)	0,6 (0,4 - 0,8)	4 (3- 6)
24 h	3	5 (5 - 8)	0,8 (0,6 - 0,9)	13 (7 - 15)
48 h	3	7 (5 - 7)	1,0 (0,5 - 1,1)	5 (3 - 8)
72 h	2	(5 - 6)	(0,9 - 1,4)	(1 - 3)
96 h	2	(5 - 6)	(0,5 - 0,8)	(1 - 2)

Supplementary table 2. Numbers are median and range of cells within the indicated immune cell populations in peripheral blood, and are given as absolute number x 10^9. N=number of animals investigated.
